# Lessons Learned From Clinical Recruitment of Older Adults With MCI for an Internet-Delivered Intervention Study

**DOI:** 10.1093/geroni/igab046.122

**Published:** 2021-12-17

**Authors:** Meghan Mattos, Mark Quigg, Carol Manning, Eric Davis, Ann Sollinger, Laura Barnes, Lee Ritterband

**Affiliations:** 1 University of Virginia, School of Nursing, Charlottesville, Virginia, United States; 2 University of Virginia, Charlottesville, Virginia, United States; 3 University of Virginia, School of Medicine, Charlottesville, Virginia, United States; 4 Carilion Clinic, Roanoke, Virginia, United States; 5 University of Virginia, School of Engineering & Applied Science, Charlottesville, Virginia, United States

## Abstract

Clinical research involving participants with mild cognitive impairment (MCI) presents challenges to recruitment that may be further compounded by concerns when delivering a behavioral intervention via the Internet. The purpose of this talk is to describe recruitment adaptations for an Internet-delivered behavioral intervention study with older adults living with MCI and insomnia. Over the course of study recruitment, unforeseen barriers to recruitment were discovered, including fewer older adults with MCI endorsing sleep concerns than expected. The most substantive changes made to improve clinical recruitment were related to eligibility criteria, yielding 50% of the overall sample. Anticipated concerns of older adults with MCI using technology or accessing the Internet were not significant barriers to recruitment. Study findings support Internet-delivered intervention use in this population, which in the context of the COVID-19 pandemic, presents a potentially efficient and effective method for recruiting and delivering behavioral interventions in this difficult-to-enroll population.

